# The gut-muscle axis: a comprehensive review of the interplay between physical activity and gut microbiota in the prevention and treatment of muscle wasting disorders

**DOI:** 10.3389/fmicb.2025.1695448

**Published:** 2025-10-30

**Authors:** Yan Xu, Benxiang He

**Affiliations:** College of Sports and Health, Chengdu Sport University, Chengdu, China

**Keywords:** skeletal muscle wasting, sarcopenia, cachexia, gut-muscle axis, gut microbiota, physical activity, short-chain fatty acids

## Abstract

Skeletal muscle wasting disorders, such as sarcopenia and cachexia, pose a significant clinical challenge. The gut-muscle axis, a bidirectional signaling network, is now understood to be a critical regulator of muscle homeostasis, with the gut microbiota functioning as a key metabolic organ. Physical activity is a cornerstone intervention, exerting benefits by directly stimulating muscle and by favorably modulating the composition and metabolic output of the gut microbiota. This review synthesizes the molecular mechanisms of muscle wasting and the pathways of the gut-muscle axis, with a specific focus on microbial metabolites like short-chain fatty acids (SCFAs). We analyze how different exercise modalities modulate this system and critically evaluate evidence from human trials. By identifying key research gaps, this review argues for a paradigm shift toward integrated, personalized interventions that combine targeted exercise with nutritional and microbial strategies to more effectively combat muscle wasting disorders.

## 1 Introduction

Muscle wasting disorders, encompassing both sarcopenia and cachexia, are progressive and debilitating syndromes that lead to the involuntary loss of skeletal muscle mass and strength (Carlier et al., 2015; Brogi et al., 2024). Sarcopenia, first defined by Rosenberg in 1989, is characterized as a geriatric syndrome marked by a progressive decline in muscle mass, strength, and physical performance, often exacerbated by aging (Wang et al., 2025; Dohertya, 2010; Cederholm et al., 2011). Its prevalence is alarmingly high, affecting 5–13% of adults aged 60–70 and rising to 11–50% in those over 80 (Wang et al., 2025; von Haehling et al., 2010; Shafiee et al., 2017), with this wide range reflecting differences in diagnostic criteria and the specific populations studied. The clinical implications are severe, including an increased risk of falls, fractures, mobility challenges, and a diminished quality of life (Wang et al., 2025; Costa et al., 2008). In contrast, cachexia is a more severe wasting syndrome associated with chronic, systemic diseases such as cancer (Mortellaro et al., 2024), heart failure (Maeda et al., 2024), AIDS (Li Y-. H. et al., 2022), and chronic kidney disease (Rahbar Saadat et al., 2025), and is marked by significant loss of both fat and fat-free mass, along with intense systemic inflammation (Wang et al., 2025; Biolo et al., 2014; Kotler, 2000).

Skeletal muscle homeostasis is governed by a delicate balance between anabolic and catabolic signaling pathways (Liu and Tang, 2022; McCarthy and Murach, 2019). While traditional therapeutic approaches have focused on nutritional support and exercise, emerging evidence points to a multi-organ communication network as a critical therapeutic target (Wang et al., 2025, 2021; Monda et al., 2017; Afzaal et al., 2022). Within this network, the human gut microbiota—a complex ecosystem of trillions of microorganisms—is now recognized as a vital player in host health, functioning as a “metabolic organ” that modulates immune responses and produces beneficial metabolites from indigestible carbohydrates (Monda et al., 2017; Afzaal et al., 2022; Zhou et al., 2024).

The reciprocal influence between the gut microbiota and skeletal muscle, termed the “gut-muscle axis” or, more specifically, the “gut microbes-muscle axis,” has been substantiated by a growing body of evidence, highlighting its potential role in the pathogenesis of muscle wasting disorders (Li G. et al., 2022; Chae and Lee, 2023; He et al., 2025; Chew et al., 2023). This review therefore synthesizes the intricate mechanisms of this axis, focusing specifically on how physical activity serves as a potent, non-pharmacological modulator of the gut microbiota to prevent and treat sarcopenia and cachexia. In doing so, we frame these components as an integrated signaling network—the gut-muscle axis—whereby physical activity serves as a primary modulator.

## 2 Molecular pathogenesis of muscle wasting: a foundation for intervention

### 2.1 The anabolic-catabolic imbalance

Muscle atrophy is a pathological state defined by a reduction in muscle fiber size and overall muscle mass, which occurs when protein degradation outpaces protein synthesis (Fanzani et al., 2012). This imbalance is regulated by an intricate network of anabolic and catabolic signaling pathways (Liu and Tang, 2022).

Catabolic signaling is primarily driven by proteolytic systems that dismantle muscle proteins (Chapela et al., 2023). The most prominent of these is the ubiquitin-proteasome system (UPS), which tags proteins with ubiquitin for targeted removal by the proteasome (Pang X. et al., 2023). In muscle atrophy, muscle-specific E3-ubiquitin ligases such as MuRF-1 and MAFbx (atrogin-1) are transcriptionally upregulated, marking contractile proteins for degradation (Bowen et al., 2015; Peris-Moreno et al., 2021). Other major proteolytic pathways include the calpain pathway and the autophagy-lysosomal pathway, which also contribute to the loss of muscle mass (Bowen et al., 2015; Triolo and Hood, 2021). A potent catabolic factor is myostatin, a member of the TGFβ family that acts as a negative regulator of muscle growth (Bonaldo and Sandri, 2013). Myostatin-induced atrophy is mediated by its capacity to block the key anabolic IGF-1-PI3K-Akt pathway and activate the transcription factor FoxO1, thereby increasing the expression of atrogin-1 (Bonaldo and Sandri, 2013; Permpoon et al., 2025; Xu et al., 2023). This direct antagonism between myostatin and the IGF-1/Akt pathway is a crucial mechanistic aspect of atrophy, as a catabolic signal actively suppresses an anabolic one, creating a powerful feedback loop that accelerates muscle loss (Huang et al., 2022a).

In contrast, muscle anabolism is driven by growth factors and hormones such as Insulin-like Growth Factor 1 (IGF-1) and insulin (Fanzani et al., 2012; Lowe, 2024). The central pathway for protein synthesis is the PI3K-Akt-mTOR pathway, which promotes myocyte proliferation, differentiation, and protein synthesis while simultaneously suppressing protein degradation (Wang et al., 2025; Lowe, 2024; Chen et al., 2025). Low circulating levels of IGF-1 have been associated with sarcopenia and other chronic diseases, highlighting the central role of this anabolic pathway in maintaining muscle mass (Fanzani et al., 2012; Nyul-Toth et al., 2025). The activation of the PI3K/Akt pathway is a critical step that promotes protein synthesis and reduces protein degradation, serving as a primary target for therapeutic interventions (Fanzani et al., 2012; He Y. et al., 2021; Verma et al., 2023) (Table 1).

**Table 1:** Anabolic and catabolic pathways involved in muscle wasting.

**Pathway type**	**Pathway/system**	**Key components**	**Role in muscle wasting**	**Ref**.
Anabolic-Catabolic Crosstalk	PI3K-Akt-mTOR	Insulin, IGF-1, PI3K, Akt, mTOR	Promotes protein synthesis, cell proliferation, and myocyte differentiation; suppresses protein degradation.	Feng et al., 2022
Anabolic-Catabolic Crosstalk	Myostatin/Akt Crosstalk	Myostatin, Akt, FoxO1, atrogin-1	Myostatin blocks the Akt pathway, leading to the activation of FoxO1 and upregulation of the catabolic ligase atrogin-1.	Murphy et al., 2022
Catabolic	Ubiquitin-Proteasome System (UPS)	Ubiquitin, Proteasome, MuRF-1, MAFbx (atrogin-1)	Marks and degrades contractile proteins and organelles, leading to cellular shrinkage and muscle atrophy.	Pang X. et al., 2023
Catabolic	Calpain Pathway	Calpains, Calpastatin	Cleaves myofibrillar proteins, disrupting the sarcomere structure. May play a dominant role in sarcopenia.	Haberecht-Müller et al., 2021
Catabolic	Autophagy-Lysosomal Pathway	Autophagosomes, Lysosomes	Bulk degradation of cytoplasmic components. May contribute to muscle atrophy in sarcopenia.	Triolo and Hood, 2021
Catabolic	Inflammatory Signaling	TNF-α, IL-6, NF-κB, STAT3	Pro-inflammatory cytokines that disrupt muscle metabolism and promote catabolism, particularly in cachexia.	Malla et al., 2022

Evidence suggests that the Calpain and Autophagy-Lysosomal pathways are suggested to play a particularly dominant role in the pathogenesis of sarcopenia compared to other muscle wasting conditions.

### 2.2 Differentiating sarcopenia and cachexia

Although both are muscle wasting disorders, sarcopenia and cachexia have distinct underlying mechanisms and clinical presentations (Wang et al., 2025; Rausch et al., 2021). This distinction is critical for tailoring effective therapeutic strategies. Sarcopenia is a muscle disease often associated with aging, and is primarily characterized by a progressive loss of muscle mass and strength without significant fat loss (Wang et al., 2025; Najm et al., 2024). While the UPS is involved, existing evidence is inconsistent, suggesting that other proteolytic pathways, particularly the calpain and autophagy pathways, may play a more dominant role in sarcopenia pathogenesis (Pang X. et al., 2023; Bowen et al., 2015). Sarcopenia is often associated with mild, or even undetectable, systemic inflammation, in contrast to the intense inflammatory state of cachexia (Wang et al., 2025; Jimenez-Gutierrez et al., 2022). This lower inflammatory burden may make sarcopenia more responsive to certain non-pharmacological interventions (Ispoglou et al., 2023; Clerton, 2025).

**Figure 1:**
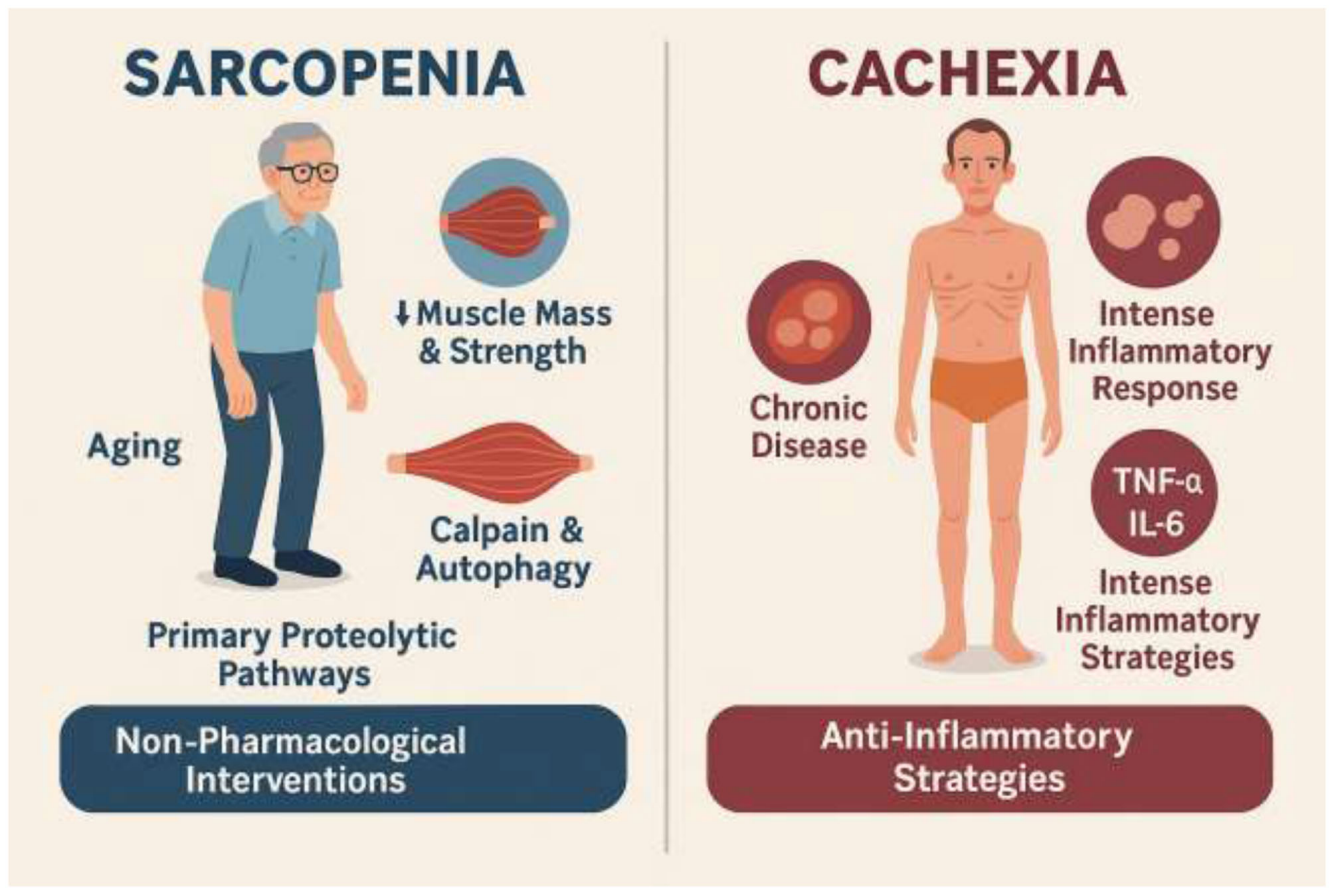
Comparative overview of sarcopenia and cachexia. Sarcopenia is an aging-associated muscle disease characterized by progressive loss of muscle mass and strength with minimal fat loss. Its pathogenesis involves calpain and autophagy pathways, with inconsistent UPS involvement, and is typically accompanied by low-grade inflammation. These features make it more responsive to non-pharmacological interventions such as exercise and nutrition. Cachexia, in contrast, is a severe wasting syndrome linked to chronic diseases (e.g., cancer, heart failure, AIDS), characterized by loss of both fat and fat-free mass, and driven by intense systemic inflammation mediated by TNF-α and IL-6. It activates proteolytic pathways including UPS, NF-κB, STAT3, and myostatin, requiring aggressive anti-inflammatory and multimodal therapeutic strategies.

Cachexia, a more severe, involuntary wasting syndrome, is typically linked to chronic diseases such as cancer, heart failure, and AIDS (Wang et al., 2025; Rausch et al., 2021). It is characterized by significant loss of both fat and fat-free mass, and is marked by an intense inflammatory response driven by pro-inflammatory cytokines such as TNF-α and IL-6 (Timmanpyati et al., 2024; He M. et al., 2021). The activation of these cytokines directly disrupts muscle metabolism and leads to the upregulation of catabolic pathways (Wang et al., 2025; He M. et al., 2021). While some signaling pathways like myostatin, NF-κB, and STAT3 are shared between the conditions, the more robust and systemic inflammatory response in cachexia necessitates more aggressive interventions (Wang et al., 2025; Ahmad et al., 2022; Malla et al., 2022; Cao et al., 2021). The distinct inflammatory profiles and primary proteolytic pathways of sarcopenia and cachexia imply that while the gut-muscle axis is relevant to both, the specific therapeutic modulation required may differ (Malla et al., 2022; Nardone et al., 2021). For example, while targeting the gut microbiota to reduce low-grade inflammation could benefit sarcopenia, a more robust anti-inflammatory strategy via the gut-muscle axis might be necessary to counteract the intense inflammatory state of cachexia (Nardone et al., 2021). This highlights the need for a personalized approach to modulating the gut-muscle axis for muscle wasting (Figure 1).

## 3 The gut-muscle axis: a bidirectional signaling network

### 3.1 Microbial metabolites as key mediators

The gut microbiota is a complex metabolic organ that transforms indigestible dietary components into a diverse array of metabolites that influence host health (Monda et al., 2017; Zhang, 2022). Among the most critical are short-chain fatty acids (SCFAs)—primarily acetate, propionate, and butyrate—produced through the anaerobic fermentation of dietary fibers (Facchin et al., 2024; Ashaolu et al., 2021). These metabolites are absorbed from the gut lumen and enter systemic circulation to modulate host metabolic responses, including those in skeletal muscle (Frampton et al., 2020; Lefevre and Bindels, 2022).

A closer examination of the individual SCFAs reveals distinct roles. In the human colon, these are typically found in a molar ratio of approximately 60:20:20 for acetate, propionate, and butyrate, respectively (Morrison and Preston, 2016).

Acetate, as the most abundant SCFA, plays a crucial role in host energy balance. It serves as a substrate for lipid synthesis and can enhance mitochondrial function and glucose metabolism in skeletal muscle, while also upregulating key genes like myoglobin and GLUT4 (Liu et al., 2025; Swalsingh et al., 2022). Furthermore, acetate can mitigate the negative effects of gut microbiota depletion on muscle development (Liu et al., 2025; Yang et al., 2024).

Propionate is primarily absorbed and utilized by the liver, where it acts as a critical substrate for hepatic gluconeogenesis. This function helps regulate blood glucose levels, which in turn improves insulin sensitivity in peripheral tissues like skeletal muscle (Monda et al., 2017; Pang R. et al., 2023).

Although less abundant, butyrate is often highlighted for its pivotal role in gut health and systemic anti-inflammatory effects. It is the primary energy source for colonocytes, enhancing the integrity of the intestinal epithelial barrier (Facchin et al., 2024; Salvi and Cowles, 2021). Importantly for muscle health, butyrate has been shown to support muscle mass preservation by suppressing inflammation and regulating autophagy, a key catabolic pathway in muscle wasting (Liu et al., 2025). This detailed understanding of the individual functions of SCFAs provides a rationale for personalized dietary and microbial interventions designed to modulate specific SCFA production for different clinical outcomes (Rauf and Khalil, 2022).

Beyond SCFAs, the gut microbiota is a critical regulator of host amino acid metabolism, which directly impacts muscle health. Gut microbes can synthesize essential amino acids and modulate the levels of circulating amino acids that serve as building blocks for muscle protein. For example, specific amino acids such as glutamine and leucine have been shown to activate the anabolic Akt/mTOR signaling pathway and upregulate the expression of myogenic factors like MyoD and myogenin, thereby promoting muscle regeneration (He et al., 2025). Furthermore, microbial metabolism of tryptophan into compounds like indolepropionic acid can exert local and systemic antioxidant effects, potentially protecting muscle from oxidative damage (Owe-Larsson et al., 2025; Jiang et al., 2022).

Secondary bile acids and vitamins, both heavily influenced by microbial activity, also act as key signaling molecules in the axis. Microbes convert primary bile acids from the liver into secondary bile acids, which can activate receptors in muscle tissue, such as TGR5, to influence energy metabolism and muscle growth (Zhao et al., 2025; Ferrell and Chiang, 2021). Similarly, the gut microbiota synthesizes essential vitamins, including B vitamins and vitamin K. These vitamins are crucial cofactors in energy metabolism and can protect muscle from damage; for instance, vitamin K has been shown to downregulate atrophy-related proteins during inflammatory states (He et al., 2025). This highlights a complex system where microbial processing of both host- and diet-derived compounds creates a pool of bioactive molecules that regulate muscle function.

However, the gut microbiota also metabolizes undigested protein. The catabolism of indigestible protein can lead to the production of potentially detrimental metabolites such as ammonia, amines, polyamines, and branched-chain fatty acids (Rodríguez-Romero et al., 2022; Torres et al., 2023; Duncan et al., 2021). This creates a paradox: while dietary protein is essential for muscle anabolism, an excess of undigested protein reaching the large intestine can promote the growth of proteolytic bacteria, which produce these potentially toxic metabolites (Ashkar and Wu, 2023; Prokopidis et al., 2021). This underscores the need for a holistic, balanced nutritional approach that includes sufficient dietary fiber to promote saccharolytic fermentation over proteolytic fermentation, rather than a single-macronutrient focus on protein alone (Table 2).

**Table 2:** Metabolites produced by microbiome and their mechanisms of action.

**Metabolite**	**Production source**	**Mechanisms of action on skeletal muscle**	**Broader physiological role**
Acetate	Fermentation of dietary fiber by gut microbes	Enhances mitochondrial respiration and glucose metabolism; upregulates myoglobin and GLUT4 genes; promotes myotube fusion (Liu et al., 2025).	Most abundant SCFA; modulates host energy metabolism and gut hormone secretion (Facchin et al., 2024).
Propionate	Fermentation of dietary fiber by gut microbes	Improves insulin sensitivity and boosts glucose uptake; exhibits anti-inflammatory properties (Liu et al., 2025).	Key substrate for hepatic gluconeogenesis; modulates blood glucose levels (Monda et al., 2017).
Butyrate	Fermentation of dietary fiber by gut microbes	Suppresses inflammation and regulates autophagy, which helps preserve muscle mass (Liu et al., 2025).	Primary energy source for colonocytes; enhances gut epithelial barrier integrity (Facchin et al., 2024).
Ammonia, Amines, Polyamines	Fermentation of undigested protein by proteolytic bacteria	Can be detrimental at high concentrations; linked to pathological states (Rodríguez-Romero et al., 2022).	Potentially toxic; an increased presence may be associated with disease (Rodríguez-Romero et al., 2022).

### 3.2 The intestinal barrier as a critical mediator

Beyond the production of metabolites, the structural and functional integrity of the intestine itself is a cornerstone of the gut-muscle axis. The intestinal epithelium forms a critical barrier that regulates the absorption of nutrients essential for muscle protein synthesis while simultaneously preventing the translocation of pro-inflammatory microbial components, such as lipopolysaccharide (LPS), into systemic circulation (Vancamelbeke and Vermeire, 2017). A compromised or “leaky” gut barrier allows for endotoxemia, which can trigger a state of low-grade systemic inflammation. This inflammation directly contributes to muscle wasting by activating catabolic signaling pathways (e.g., NF-κB) in skeletal muscle and promoting anabolic resistance. Therefore, a healthy intestinal barrier is essential for maintaining muscle homeostasis, acting as a gatekeeper that translates gut health into systemic metabolic and inflammatory balance.

### 3.3 Bidirectional signaling via hormones, cytokines, and extracellular vesicles

The gut-muscle axis is maintained by a complex, bidirectional flow of information mediated by hormones, cytokines, and extracellular vesicles (EVs). Gut microbes stimulate intestinal enteroendocrine cells to secrete hormones such as glucagon-like peptide-1 (GLP-1) and ghrelin, which enter circulation and influence muscle glucose uptake and anabolism. In the reverse direction, exercising muscle releases signaling molecules known as myokines (e.g., IL-6, IGF-1), which can travel back to the gut and modulate microbial composition and intestinal function (He et al., 2025; Leeuwendaal et al., 2021; Everard and Cani, 2014). This bidirectional hormonal and cytokine crosstalk ensures that the metabolic state of the muscle is communicated to the gut and vice-versa.

More recently, extracellular vesicles (EVs) derived from gut microbes have been identified as novel mediators in this axis. These lipid-bilayer vesicles can transport a wide array of bioactive cargo—including nucleic acids, proteins, and metabolites—from the gut lumen into systemic circulation, eventually reaching peripheral tissues like skeletal muscle. It has been demonstrated that microbial EVs can directly influence insulin signaling and glucose uptake in muscle cells (He et al., 2025; Sun et al., 2023; Kumar et al., 2024; Wu et al., 2024). EVs thus represent a direct transport mechanism, allowing microbial components to exert functional effects on muscle physiology far from the gut itself.

## 4 Physical activity as a therapeutic modulator of the gut-muscle axis

### 4.1 Exercise-induced changes in gut microbiota composition and diversity

As a key behavioral input, physical activity does not act on muscle in isolation; instead, it serves as a powerful regulator of the entire gut-muscle axis. Physical activity is a powerful environmental factor that can reshape the gut microbiota. Numerous studies have shown that exercise enhances microbial diversity and enriches the microflora with beneficial species (Lapauw et al., 2024; Dziewiecka et al., 2022; Campaniello et al., 2022). Athletes and active individuals generally exhibit greater microbial biodiversity and a higher abundance of SCFA-producing bacteria, such as *Faecalibacterium prausnitzii*, than their sedentary counterparts (Dziewiecka et al., 2022; Sohail et al., 2019). Longitudinal studies have further confirmed that a transition from a sedentary to an active lifestyle can reduce disease-related bacteria and increase health-associated taxa (Boytar et al., 2023; Lelonek et al., 2025). This positive modulation of the gut microbiota, which includes improved barrier function and reduced systemic inflammation, is a key mechanism by which exercise promotes overall health and mitigates disease (Monda et al., 2017; Dmytriv et al., 2024).

**Figure 2:**
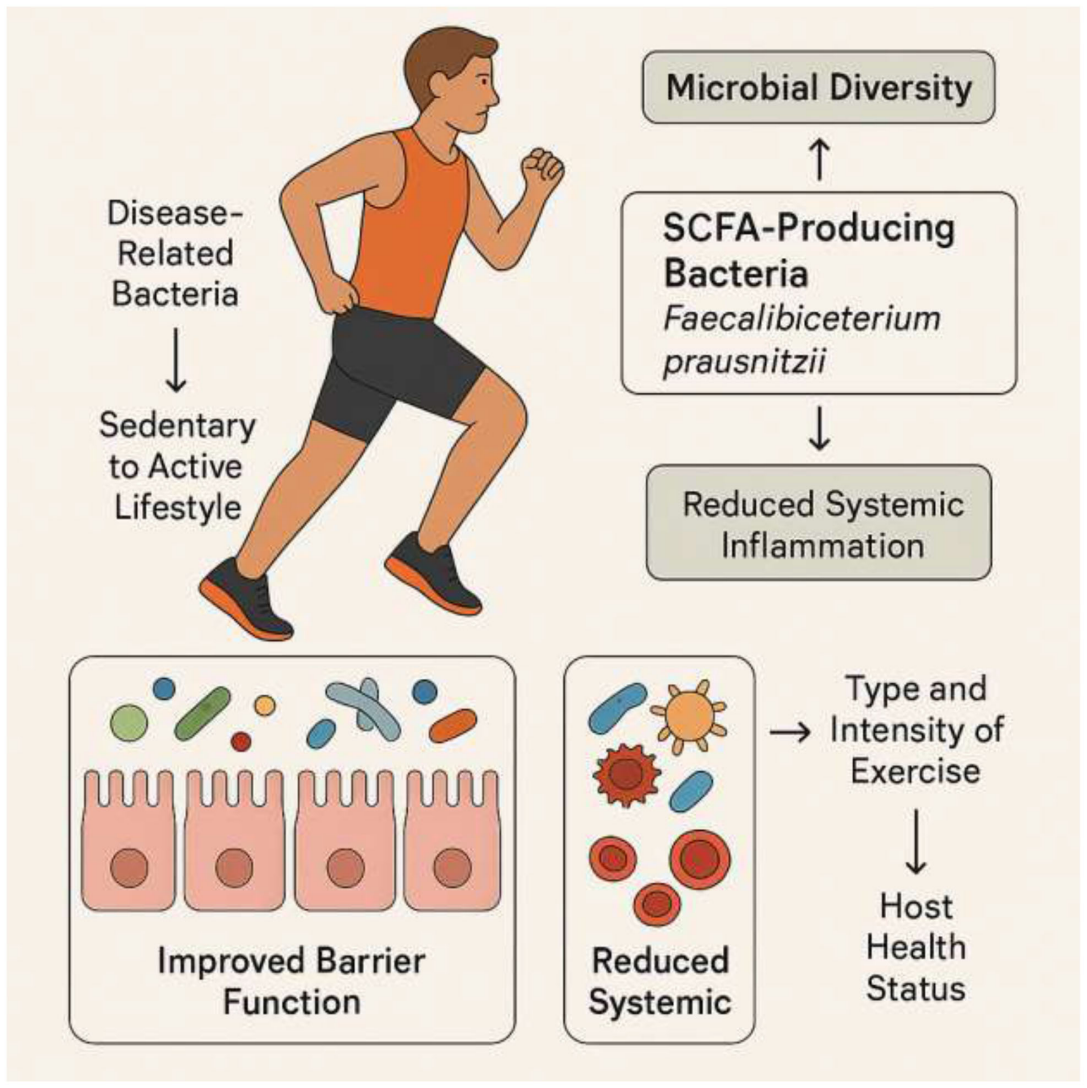
Exercise-mediated modulation of gut microbiota. Transitioning from a sedentary to an active lifestyle enhances microbial diversity and increases the abundance of SCFA-producing bacteria such as *Faecalibacterium prausnitzii*. These changes improve gut barrier function and reduce systemic inflammation, contributing to overall health. The effects of exercise vary with type, intensity, and host health status: moderate-intensity continuous training (MICT) enriches species like *Lachnospira eligens* and *Clostridium Cluster IV*, whereas high-intensity interval training (HIIT) promotes other butyrate-producing taxa from *Erysipelotrichales* and *Oscillospirales*. Obesity and metabolic diseases may blunt these beneficial responses, highlighting the importance of personalized exercise prescriptions.

However, the effects of exercise on the gut microbiota are not universal; they are contingent on several variables, including the type and intensity of exercise, as well as the host's health status (Bonomini-Gnutzmann et al., 2022; Cataldi et al., 2022). For example, a six-week endurance exercise intervention increased fecal SCFAs in lean but not obese participants (Allen et al., 2018). This suggests that obesity-related dysbiosis or inflammation may blunt the gut's metabolic response to exercise. A separate study on individuals with type 2 diabetes found that different exercise intensities increased the abundance of distinct butyrate-producing species (Torquati et al., 2023). Moderate-intensity continuous training (MICT) led to a higher relative abundance of *Lachnospira eligens* (Torquati et al., 2023) and *Clostridium Cluster IV* (Torquati et al., 2023), while high-intensity interval training (HIIT) promoted other butyrate-producers from *Eryspelothrichales* and *Oscillospirales* (Torquati et al., 2023). This finding is significant because it indicates that different exercise prescriptions can specifically target and modulate distinct microbial communities and their functions (Figure 2 and Table 3).

**Table 3:** Impact of exercise on microbiome.

**Exercise modality**	**Study population**	**Key microbiota changes**	**Changes in SCFA production**	**Further description**
Endurance	Lean, previously sedentary	Increased overall microbial diversity (Allen et al., 2018). Increased relative abundance of	*Akkermansia* and a decrease in *Proteobacteria* (Erlandson et al., 2021).	Increased fecal SCFAs in lean participants, but not in obese participants (Allen et al., 2018).
Moderate-Intensity Continuous Training (C-MICT)	Low active people with Type 2 Diabetes	Higher post-exercise abundance of *Bifidobacterium, A. municiphila*, and butyrate-producers like *Lachnospira eligens* and *Clostridium Cluster IV* (Torquati et al., 2023).	No significant change in fecal SCFAs compared to HIIT group (Torquati et al., 2023)	
High-Intensity Interval Training (C-HIIT)	Low active people with Type 2 Diabetes	Higher post-exercise abundance of other butyrate-producers from *Eryspelothrichales* and *Oscillospirales* (Torquati et al., 2023).	No significant change in fecal SCFAs compared to MICT group (Torquati et al., 2023).	
Resistance and Aerobic (Combined)	Younger and older individuals	Significant modifications of fecal microbiota composition (Burtscher et al., 2022). Increased representation of	*Bifidobacteria* and *Faecalibacterium prausnitzii* (Burtscher et al., 2022).	SCFA changes were noted in master athletes compared to sedentary controls (Burtscher et al., 2022).

### 4.2 The differential impact of exercise modalities on muscle and gut homeostasis

Different forms of physical activity elicit distinct physiological adaptations in muscle, and mounting evidence suggests a similar specificity in their effects on the gut microbiome.

- **Resistance Training (RT):** This modality is a potent stimulus for increasing muscle mass and strength, primarily through muscle hypertrophy and neural adaptations (McLeod et al., 2024; Alix-Fages et al., 2022). RT prescription variables like volume (number of sets) and load are key determinants of its effectiveness (McLeod et al., 2024).

While RT's influence on gut microbiota is a developing area of research, emerging evidence suggests its effects are distinct from that of endurance training. Unlike the consistent increases in microbial diversity seen with aerobic exercise, RT appears to exert a more targeted influence on microbiota composition (Wegierska et al., 2022; Varghese et al., 2024; Polo-Ferrero et al., 2025; Zhong et al., 2022; Cullen et al., 2024). Recent studies have demonstrated that structured RT can increase the abundance of beneficial, SCFA-producing genera such as Roseburia and Faecalibacterium, particularly in individuals who show significant strength gains (Meiners et al., 2024; Zhang et al., 2024). Furthermore, some research indicates RT may improve gut barrier integrity by increasing the metabolic capacity of the microbiome to produce mucin and decreasing serum zonulin, a marker associated with intestinal permeability (Di Vincenzo et al., 2024; Dmytriv et al., 2024). These findings suggest that RT's primary impact may be on modulating specific microbial functions related to gut health and anti-inflammatory pathways, rather than broad changes in diversity.

- **Endurance Training (ET):** Classically performed at a low load for a long duration, ET enhances cardiorespiratory fitness, promotes mitochondrial biogenesis, and increases capillary density (Hughes et al., 2018; Mølmen et al., 2025). These adaptations improve the muscle's ability to utilize oxygen and delay fatigue (Hughes et al., 2018). As a potent modulator of the gut microbiome, endurance training is a potent modulator of the gut microbiome, consistently shown to enhance microbial diversity and metabolic function (Clauss et al., 2021). Mechanistically, ET can increase gut motility and blood flow, creating a favorable environment for the proliferation of SCFA-producing bacteria (Hawley et al., 2025). These microbial shifts are directly linked to higher circulating levels of butyrate, which confers systemic anti-inflammatory benefits and serves as an energy source for colonocytes. This enhancement of SCFA production is a cornerstone of how endurance exercise translates into improved metabolic health and supports the gut-muscle axis (Martín et al., 2023; Singh et al., 2022).

The combination of RT's direct hypertrophy benefits and ET's systemic metabolic and microbial benefits suggests that concurrent training might be the most effective strategy. Resistance training directly stimulates protein synthesis and combats muscle atrophy (Gedara and Othalawa, 2023), while endurance training improves mitochondrial function and cardiorespiratory fitness (Hughes et al., 2018). Endurance training has also been shown to modulate the gut microbiota and SCFA production (Sohail et al., 2019; Huang et al., 2022b). A combined approach could leverage both the direct mechanical stimulus of RT and the systemic, metabolic, and microbial benefits of ET. The ongoing DEMGUTS study (NCT06545123) is a prime example of a clinical trial designed to test this very hypothesis, directly comparing the effects of aerobic, resistance, and concurrent exercise on gut microbiota and physical outcomes in older adults with sarcopenia (Merelim et al., 2025).

A critical clinical consideration is the reversibility of exercise-induced benefits. The positive effects of exercise on muscle strength, endurance, and gut microbiota composition are largely reversed when training ceases (Bonomini-Gnutzmann et al., 2022; Allen et al., 2018). This underscores a fundamental principle: exercise must be a sustained, lifelong intervention to combat the progressive nature of sarcopenia and chronic disease-related muscle wasting (Chen, 2024). This shifts the clinical focus from short-term programs to promoting long-term behavioral change and adherence.

## 5 Clinical and translational perspectives

### 5.1 Evidence from human studies and research challenges

Observational studies in elderly populations have consistently shown significant differences in the gut microbiota between individuals with and without sarcopenia (Lapauw et al., 2024; Liu et al., 2021). Sarcopenic patients have been found to have lower microbial alpha-diversity (richness and diversity) compared to those with preserved muscle status (Lapauw et al., 2024). The microbial composition of sarcopenic individuals also clusters differently, with specific genera, including *Blautia, Lachnospiraceae*, and *Subdoligranulum* (Liu et al., 2021), identified as having potential diagnostic value for the disease (Zhou et al., 2023). Metabolomics analysis further links sarcopenia to significant alterations in 172 metabolites and pathways, including butanoate metabolism, which is a key SCFA pathway (Zhou et al., 2023). However, the field faces significant challenges that hinder direct translation of these findings into clinical practice. The heterogeneity of study designs, exercise prescriptions, and reporting methods complicates a direct meta-analysis of the data (Lapauw et al., 2024). Many studies are cross-sectional, which makes it impossible to establish causality—it is unclear if dysbiosis causes muscle wasting or vice versa (Lapauw et al., 2024). Furthermore, there is no universally accepted “sarcopenia-specific GM signature,” and findings are often conflicting (Lapauw et al., 2024).

### 5.2 Future directions and personalized medicine

Overcoming the aforementioned challenges is crucial for translating scientific findings into clinical practice. The progression of research in this field is a key narrative, marked by a shift from broad observational studies to targeted, hypothesis-driven longitudinal clinical trials. There is an urgent need for uniformly designed trials with large sample sizes, standardized exercise prescriptions, and clear, defined core outcome sets (Lapauw et al., 2024).

Due to the multifactorial nature of muscle wasting, the most promising therapeutic strategies will likely be multi-modal, combining exercise with targeted nutritional support and microbial modulation (Das et al., 2024). The ultimate goal is to move toward a personalized precision medicine paradigm. The future trajectory of the field will involve leveraging emerging technologies like multi-omics (genomics, transcriptomics, metabolomics) (Mohr et al., 2024) and artificial intelligence to enable the dynamic monitoring and real-time modulation of microbial activity and its impact on muscle health (Wang et al., 2023). This integrated approach will allow clinicians to design individualized exercise and nutritional prescriptions based on a patient's unique biological profile to optimize health outcomes.

## 6 Conclusion

Skeletal muscle wasting disorders are complex conditions driven by an imbalance of catabolic and anabolic signaling. A growing body of evidence has established the existence of a bidirectional gut-muscle axis, where the gut microbiota, through the production of key metabolites like SCFAs, plays a direct and profound role in regulating muscle health, metabolism, and inflammation. Physical activity is a powerful, non-pharmacological modulator of this axis, influencing microbial composition, diversity, and metabolic output in a manner dependent on the exercise modality, intensity, and host factors. While the field has progressed significantly from observational studies to interventional trials, key challenges remain, including a lack of standardized research and the heterogeneity of findings. The future of therapeutic intervention for sarcopenia and cachexia lies in a shift toward personalized, multi-modal strategies that leverage the principles of the gut-muscle axis. By integrating exercise, nutrition, and microbial modulation with advanced technologies like multi-omics and artificial intelligence, the scientific community can move closer to developing truly effective, individualized therapies to preserve muscle function and enhance the quality of life for millions of individuals affected by these debilitating disorders.
